# Deep or Shallow, It's up to You

**DOI:** 10.1055/s-0042-1756339

**Published:** 2023-12-21

**Authors:** Kun Hwang

**Affiliations:** 1 Department of Plastic Surgery, Armed Forces Capital Hospital, Bundang-gu, Seongnam-City, Gyeonggi-do, Republic of Korea; 2 Ewha Medical Academy, Ewha Womans University Medical Center, Seoul, Republic of Korea

**Figure:**
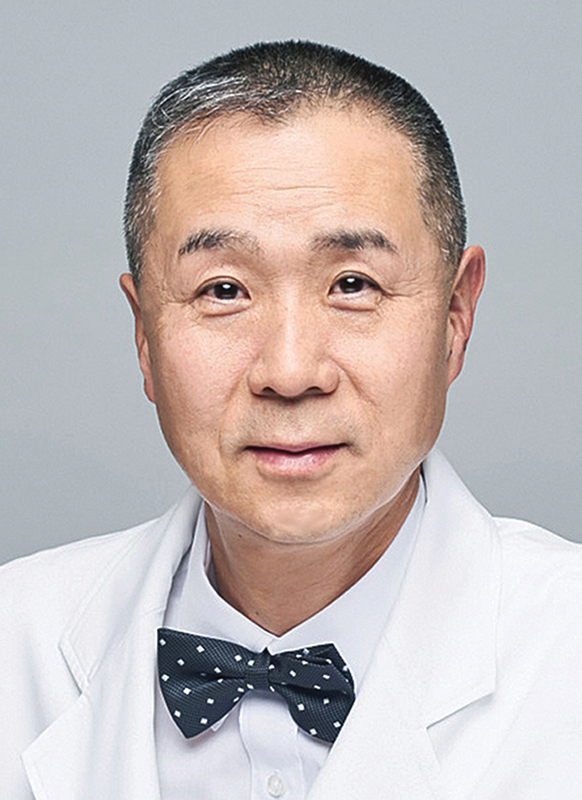
Kun Hwang, MD, PhD

We use surgical scissors to undermine subcutaneous tissue or remove a mass. Scissor blades are curved or straight. When the bows or rings opposite to the joint are closed, the sharpened edges slide against each other, resulting in tissue dissection. In some types of scissors, like facelift scissors, the sharpened lateral edge dissects the tissue when the joint is opened. In spring forceps, small scissors used in microsurgery, the handles end in flat springs connected with a pivot joint. When the handles are pressed together, the cutting action is achieved.

Personally, small curved blunt tip scissors are my favorite type for subcutaneous dissection. During dissection of a vessel-rich area, I hope I have my eye on the tip of the scissors. Sometimes I think that this small pair of scissors is a part of my hand or that the scissors have their own personality. I imagine that this pair of scissors will have a heart-to-heart with me in the operating theater, like dialogues in a movie or play.

**Fig. 1:**
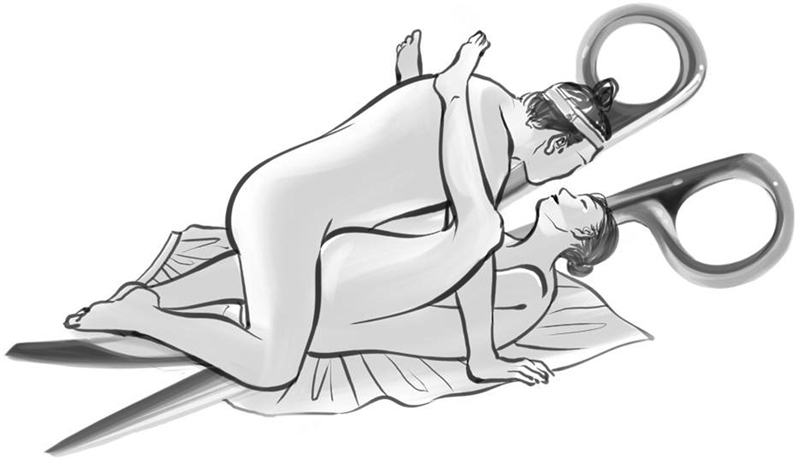
“Scissors” written by Heo Nanseolheon (  ). Illustration by Hye Won Hu, MFA, Division of Biomedical Art, Incheon Catholic University Graduate School, Incheon, Korea.

Last year, when I had a conference in Gangneung, I visited the site of birthplace and museum of Heo Nanseolheon (  , 1563–1589), a talented female poet of the Joseon dynasty (1392–1910) of Korea. Among her many beautiful poems, a poem entitled “Scissors (  )” intrigued me ( Fig. 1 ).

Scissors
We agreed to put our waists together (  )
I tenderly raised my legs (  )
I'll shake back and forth (  )
Deep or shallow, it's up to you (  )

In her era, all women were required to know how to cut out a dress. In this poem, the scissors correspond to a woman narrator. Most literary critics say that the poet metaphorically expressed the physical affair of a man and woman using the cutting mechanism of scissors.

However, from the viewpoint of a surgeon, it is admirable that she described the morphology of curved scissors (raised the legs) and the sliding of the edges at the joint (two waists together). Furthermore, she emphasized that the amount of dissection in one stroke is solely dependent upon the operator (deep or shallow, it's up to you).

At the age of 15, Heo Nanseolheon married a mediocre official. He ignored his talented wife and indulged in an extramarital affair with a young maiden. Heo Nanseolheon had two children, but lost one after another due to illness. In the Joseon era, when there was a tendency to look down upon women based on Confucianism, poetry written by women could not be allowed, and women poets were criticized simply for writing poetry. Despite this poor environment, she left 200 beautiful manuscripts before she died at the age of 26. Her poems were published after her death by her brother Heo Gyun (  ).

During dissection, I spread or close the joint of “my” scissors. They are shaking back and forth at the joint. However, I am very careful to put the tip into the soft tissue in a vessel-rich area. Deep or shallow, it's up to me!

